# The Improved Biological Performance of a Novel Low Elastic Modulus Implant

**DOI:** 10.1371/journal.pone.0055015

**Published:** 2013-02-21

**Authors:** Lei Shi, Lei Shi, Ling Wang, Yonghong Duan, Wei Lei, Zhen Wang, Jing Li, Xiangli Fan, Xiaokang Li, Shujun Li, Zheng Guo

**Affiliations:** 1 Institute of Orthopaedics, Xijing Hospital, Fourth Military Medical University, Xi'an, China; 2 Institute of Implantation, Stomatological Hospital, Fourth Military Medical University, Xi'an, China; 3 Institute of Health Statistics, Fourth Military Medical University, Xi’an, China; 4 Institute of Metal Research, Chinese Academy of Sciences, Shenyang, China; University of Zurich, Switzerland

## Abstract

**Background:**

The mismatch of the elastic modulus between implants and bone tissue can lead to stress shielding, bone resorption and poor osseointegration. Compared with normal bone tissue, this problem is much more serious in osteoporosis. The purpose of this study was designed to find out whether the novel Ti-24Nb-4Zr-7.9Sn (TNZS) implant with low elastic modulus and high strength was suitable for biomedical material, especially in osteoporosis.

**Methodology:**

*In vitro* study, the viability and Alkaline phosphatase (ALP) activity of osteoblasts on the TNZS and Ti-6V-4V (TAV) were observed. *In vivo* study, 30 adult female New Zealand rabbits were selected and divided randomly into two groups: sham-operation (SHAM, n = 6) and ovariectomised in combination with methylprednisolone treatment (OVX+MP, n = 24). Two implants were then placed in the tibia of each OVX + MP group rabbit, one in each side (left: TAV; right: TNZS). The OVX + MP group rabbits were sacrificed at 4 and 12 weeks after the implantation. The osteoporotic bone responses to the TNZS and TAV implants were evaluated by pull-out test, Micro-CT analyses and histological observation.

**Principal Findings:**

Compared with the TAV group, the TNZS group showed a significant increase (P<0.05) in cell viability and ALP activity, new bone formation and pull-out force.

**Conclusions:**

The novel TNZS implants show good biological performance both *in vitro* and *in vivo*, which suggests that the alloys are suitable for biomedical applications, especially in osteoporosis.

## Introduction

Since the early 1930s when a stainless steel implant was first used in surgery, Vitallium, Co-base alloys, and titanium (Ti) or Ti-based alloys have been developed for biomedical applications [1]. Moreover, Ti and its alloys have become one of the most attractive classes of biomedical implant materials, due to their more excellent corrosion resistance to biological environments and mechanical properties, higher strength to weight ratio and stronger adhesion to bone tissue, compared to various other metallic biomaterials [2]–[4].

The first generation orthopaedic *α*+*β* titanium alloys like Ti–6Al–4V (TAV) is being widely used, and has gotten good clinical results. However, the elastic modulus of TAV (110 GPa) used in clinic is much higher than human cancellous bone (<3 GPa) or compact bone (12–17 GPa) [5]. This large elastic modulus mismatch between implants and the surrounding human bone has been identified as a major cause of stress shielding, bone resorption and implant loosening [6], [7]. Moreover, the moduli mismatch leads to excessive micro-motion between implants and bone, which inhibits bone formation and promotes fibrous tissue ingrowth, thereby preventing the osseointegration of the implant [8]. Compared with normal bone tissue, the moduli mismatch is more serious in osteoporosis with anticipated high rates of implant loosening and failure. Thus, a novel implant with ultra-low elastic modulus and high strength is needed.

With developing of material science, the second generation *β* Ti-alloys have been invented for orthopaedic application. With low-modulus, *β* Ti-alloys can minimize the “stress shielding” effect caused by the modulus mismatch between implant and bone, thus it may be an ideal orthopaedic alloy [2]. Ti-Ni alloy has been considered for implantation material in last two decades, for its properties of unique shape-memory effect, super-elasticity and high damping [3]. However, the release and accumulation of Ni ions are thought to be a potential risk, which may be toxic to cell, influence gene expression and cholesterol metabolism and cause allergy and carcinogenicity. Thus, Ni-free Ti-alloys has been widely studied in recent years, especially the Ti-Nb alloys which have gaining increasing attraction for its potential as implantation material [9]–[11]. Many developed promising biomedical Ti-Nb alloys, such as Ti-Nb-Zr and Ti-Nb-Zr-Ta, show significant improvements in elastic modulus, strength and non-toxic composition compared to previous generation alloys, such as TAV and stainless-steel. Recently, another novel Ti-Nb alloy, Ti-24Nb-4Zr-7.9Sn (TNZS), has been developed by the Institute of Metal Research Chinese Academy of Science (PCT/CN2004/001352) [12]. After ageing treatment, the novel Ti-Nb alloy shows the satisfactory balance between low elastic modulus (42 GPa) and high strength (800–900 MPa) [13]. Theoretically, the advantageous properties of this novel alloy are suitable for medical processes, and it is expected to favour new bone formation and suppress bone resorption for long term implantation, especially in osteoporosis.

Thus, the purpose of this study was to evaluate the biological performance of the novel TNZS, both *in vitro* and *in vivo*, and determine whether it is suitable for biomaterial applications, especially in osteoporosis.

## Materials and Methods

### Ethics statement

This investigation followed international guidelines (Guide for the Care and Use of Laboratory Animals) [14] for protection of animals and was approved by the ethics committee of the Fourth Military Medical University. Sample preparation.

Ti-24Nb-4Zr-7.9Sn (TNZS) alloy and Ti-6Al-4V (TAV) alloy were provided by the Institute of Metal Research Chinese Academy of Sciences (Shenyang, China). The chemical composition of TNZS is shown in Table 1 [13]. TNZS and TAV samples with a diameter of 14.5 mm and a thickness of 1 mm were used to analyse the surface characterisation and evaluate cell biocompatibility. Cylindrical implants with an external diameter of 2 mm and a length of 6 mm were used in the animal experiment [15]. All samples were cleaned ultrasonically, rubbed with 1,200-grit silicone paper to produce a uniform surface roughness, and then sterilised in an autoclave.

**Table 1:** Chemical composition of the Ti–Nb–Zr–Sn alloy (wt. %).

Ti	Nb	Zr	Sn	O	N	H
Balance	24.1	3.92	7.85	0.11	0.008	0.006

### Surface Properties

The surface roughness was quantified using a surface profilometer (Time Group Inc., TR240). The arithmetical means (Ra) of the surface roughness were assessed.

### Cell Viability

#### Osteoblast Cell Culture

Rabbit calvaria osteoblast cells were isolated from the calvarial bone of a 15-day-old rabbit according to an established protocol [16]. The cells were cultured at 37°C in a humidified atmosphere of 5% CO_2_, in 50 cm^2^ flasks containing 5 ml Dulbecco’s Modified Eagle Medium (DMEM; Gibco) with 10% foetal bovine serum (FBS; Gibco).

### Mtt Assay

The cell viability was evaluated by a quantitative colorimetric test, the methylthiozol tetrazolium (MTT) test, which characterises cellular metabolism and cell viability [17]. Osteoblasts were seeded at a density of 20,000 cells/cm^2^ onto each disk placed in a standard 24-well tissue culture plate. Cell cultures from days 1, 4, and 7 after plating were incubated with MTT solution (5 mg/mL, Sigma-Aldrich) for 4 h at 37°C. Six disks from each group were measured at each time point. The optical density (OD) was measured at 570 nm using a 96-well enzyme immunoassay analyzer (Bio-TEK, ELX800).

### Alkaline Phosphatase (alp) Activity

For determination of the activity of alkaline phosphatase, the osteoblastic cells were cultured on discs for 1, 4 and 7 days. At each time point, the discs were washed twice with PBS, and immersed in 1.5 M Tris-HCl, pH 10.2 containing 1 mM ZnCl_2_, 1 mM MgCl_2_ and 1% Triton X-100 at 4°C for 12 hours before the ALP tests. After clarifying the cell lysates by centrifugation, ALP activity was assessed by measuring the release of p-nitrophenol spectrophotometrically at 405 nm by ELX800 enzyme immunoassay analyzer.

### Animal Surgery And Care

Thirty adult female New Zealand rabbits of 4.1±0.35 kg in weight were selected for this study. The rabbits were housed in individual cages made of stainless steel (0.49 m wide ×0.61 m deep ×0.48 m high and made of stainless steel), and kept in a 12 h light/dark cycle in a temperature-controlled (22±1°C) designated animal room. The rabbits were divided randomly into two groups: sham-operation (SHAM, n = 6) and ovariectomised with methylprednisolone treatment (OVX+MP, n = 24). Two weeks after bilateral ovariectomy, the rabbits in the OVX+MP group were injected intramuscularly with methylprednisolone succinate dissolved in 0.9% benzyl alcohol at a dosage of 1 mg/kg/day for 8 consecutive weeks; the rabbits in the SHAM group were injected with 0.9% benzyl alcohol [18]. All of the animals were assessed for bone mineral density (BMD) using dual-energy X-ray absorptiometry (DEXA Lunar; GE Healthcare) to confirm the usefulness of the OVX+MP group as an osteoporosis model. After confirming that the osteoporotic model was successfully established in the OVX+MP group, the implantation was performed.

All of the surgical procedures were performed in aseptic conditions and under general anaesthesia. A small 5-mm skin incision was made to provide access to the bone. Implantation sites were prepared by drilling a cavity with low rotational speed, while profuse irrigation with sterilised physiologic saline was maintained [19]. Two implants were placed in the tibia of each animal, one in each side (left: TNZS, right: TAV). After the operation, the animals were allowed to bear their full weight and received antibiotics (penicillin, 40,000 U/d) for 3 days. The OVX group rabbits were sacrificed in two groups at 4 and 12 weeks after the operation. All of the tibias were harvested and stripped of overlying skin and muscle tissue. Half of them were used for pull-out testing immediately; others were rinsed in sterile saline and transferred to 10% neutral buffered formalin.

### Pull-Out Strength Test

The bone and soft tissues that had formed on top of the implants were carefully removed before the test. Six samples from the TNZS and TAV groups were measured at 4 and 12 weeks, respectively. Axial pull-out was performed at 1.0 mm/min displacement. A universal testing instrument (Shimadzu, AGS-10kNG) was used to measure the peak value of the pull-out force (Fmax).

### Micro-Ct Analysis

The specimens with inserted implants were examined by micro-CT scanner (Explore Locus SP, GE Healthcare) using a voxel size of 14-μm in all three axes. Six samples in both the TNZS and TAV groups were measured at 4 weeks and 12 weeks, and the images of the bone and implants were then obtained and reconstructed. A tube area with a diameter of 1 mm around the implants was defined as the region of interest (ROI), where the bone volume (BV) and tissue mineral density (TMD) were calculated.

### Histological Observation

After being fixed in 10% formalin for 1 week, the specimens were immersed into 70%, 80%, and 90% n-butanol, alcohol and dimethyl benzene subsequently and for 2 h, respectively. After being embedded in plastic liquid (butyl benzene o-dicarboxylate, butyl methacrylate, benzoylperoxide), the specimens were cooled at −20°C to become hard solids. Thick sections (50 to 100 μm) were cut with a band saw (Leica SP1600, Germany) and stained with Stevenel’s blue and van Gieson’s picrofuchsin. Qualitative morphological assessment was carried out using LEICA DM LA image analysis software. A thorough microscopic analysis was performed on sections using transmitted light microscopy (Leica-LA) combined with a digital camera (Pixera Pro600cl, USA).

### Statistics

The data were presented as the mean ± SD and analysed by *t*-test with a significance level of 0.05. Statistical analyses were performed using SPSS 16.0 software.

## Results

### Surface Properties

From the roughness measurements, the Ra values ranged between 0.1 and 0.3 μm. The Ra of TNZS (0.238±0.041 μm) was similar to the Ra of TAV (0.211±0.032 μm) (P>0.05, n = 6).

### Cell Viability And Alp Activity

**Figure 1:**
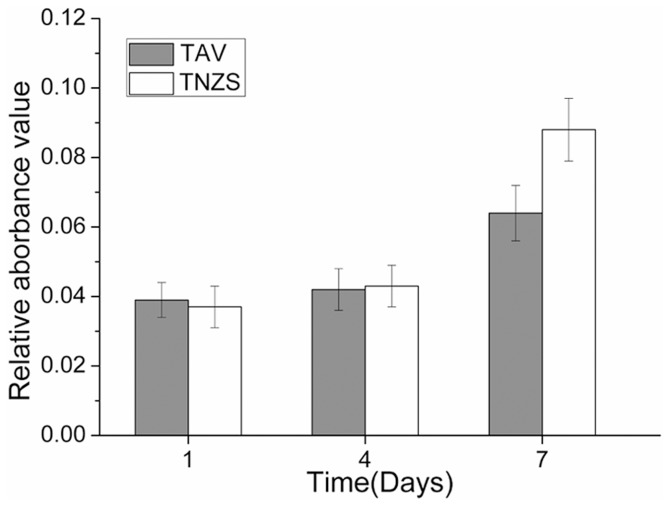
The viability of Osteoblast. The viability of osteoblast on the surface of TAV and TNZS at day 1, day 4, and day 7. At day 7, increased values of OD on TNZS were statistically different from the TAV specimens (p<0.05).

From day 1 to day 7, the osteoblast viability indicated by the MTT assay continuously increased in both the TNZS and TAV groups (Fig. 1). There was no significant difference between the two groups at day 1 and day 4 (P>0.05); however, significantly higher absorbance was detected in the TNZS group at day 7 compared to the TAV group (P<0.05).

**Figure 2:**
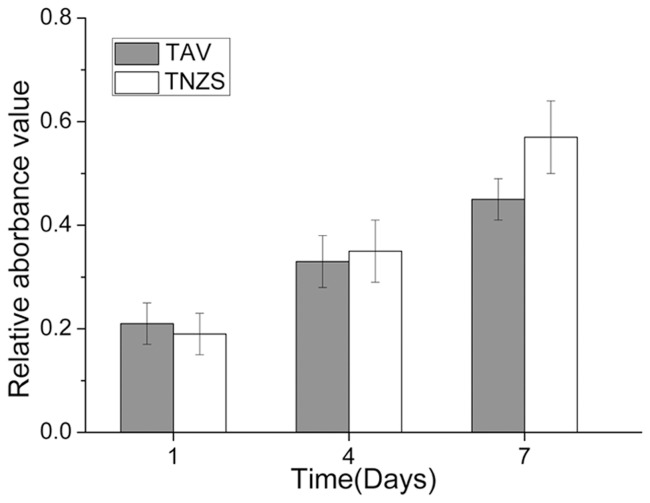
The ALP activity of Osteoblast. The ALP axtivity of osteoblast on the surface of TAV and TNZS at day 1, day 4, and day 7. At day 7, increased values of OD on TNZS were statistically different from the TAV specimens (p<0.05).

Similar to the viability of osteoblast cells, the ALP activity increased from day 1 to 7 in both groups (Fig. 2). There was no statistical difference on ALP activity between the two groups at before day 4 (P>0.05); but at day 7, the ALP activity in TNZS group was significantly higher than that of TAV group (P<0.05).

### Bmd

A rabbit osteoporosis model was successfully established in the OVX+MP group by OVX and injection with methylprednisolone. The BMD of the lumbar spine was determined (mean ± SD). The BMD of the OVX+MP group (205±28 mg/cm2) was decreased 32.1% compared to the SHAM group (302±37 mg/cm2), and the difference was significant (P<0.01).

### Pull-Out Test

**Figure 3:**
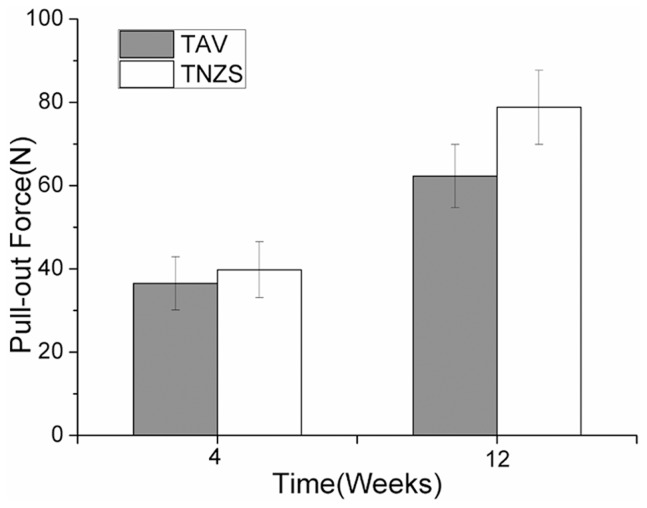
The biomechanical test. The Max Pull-out force values of the TAV and TNZS groups at 4 and 12 weeks, n = 6.

The pull-out strength of the implants in the tibia of rabbits at 4 weeks and 12 weeks was measured, and the results are shown in Fig. 3. There was no significant difference between the TNZS (36.3±4.6 N) and TAV groups (33.7±4.2 N) at 4 weeks; however, a higher pull-out strength was detected in the TNZS group (72.6±9.8 N) compared to the TAV group (58.7±8.6 N) at 12 weeks (P<0.05).

### Micro-Ct Analysis

**Figure 4:**
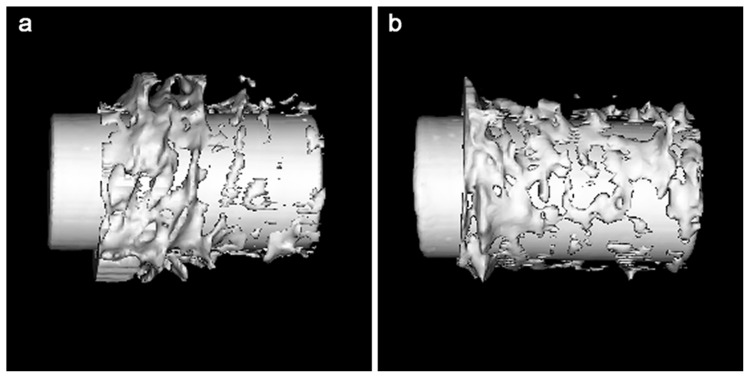
Micro-CT analysis of implant-bone interface. a and b separately show the implant-bone interfaces in the TAV group and the TNZS group at 12 weeks. White colour represents bone tissue, and light grey indicates the implant. (Resolution 21 μm, 2048×2048).

The results of the Micro-CT are shown in Table 2. At 4 weeks, the bone volume (BV) and tissue mineral density (TMD) were almost the same in the TNZS and TAV groups. However, at 12 weeks, the TNZS group had visually greater bone formation than that in the TAV group in the ROI (Fig. 4), and the BV and TMD were significantly increased in the TNZS group compared to the TAV group (P<0.05).

**Table 2:** Three dimensional parameters of the ROI in the two groups (n = 6, *x* ± s).

	TAV	TNZS	*t*	*P*
4 weeks (n = 6)				
BV (mm^3^)	9.15±1.56	10.82±1.73	−1.756	0.110
TMD (mg/cc)	574.58±57.08	625.47±64.39	−1.449	0.178
12 weeks (n = 6)				
BV (mm^3^)	15.26±2.27	18.92±2.73*	−2.525	0.031
TMD (mg/cc)	861.25±80.08	981.37±90.26*	−2.438	0.035

ROI indicates region of interest, BV bone volume, TMD tissue mineral density.

* significant differences were found compared with TAV group (P<0.05).

### Histological Observations

**Figure 5:**
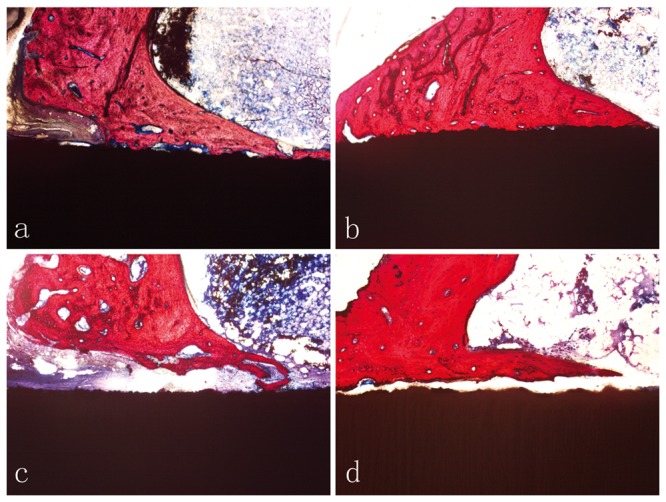
Histological observation of implant-bone interface. a and c represent the TNZS group and the TAV group at 4 weeks post-operation; b and d represent the 12 weeks after post-operation. Red colour represents bone tissue, and black colour indicates the implants. (Ponceau ×50).

The histological evaluation showed no inflammatory response in either group, and drilling debris and fractured bone particles were absent. Newly formed bone occurred around the implants, and a bone bridge appeared in the bone marrow cavity in both groups. At 4 weeks, fibrous tissue and osteoid were observed at the bone-implant interface in both the TNZS and TAV groups. At 12 weeks, the fibrous tissue and osteoid disappeared and were replaced by new bone tissue in the TNZS group, but the fibrous tissue still existed in the TAV group; moreover, direct bone-to-implant contact was observed in the TNZS group (Fig. 5).

## Discussion

The mismatch of the elastic modulus between implants and bone tissue has been identified as a major cause of stress shielding, bone resorption and implant loosening [5],[6]. In osteoporosis, the problem is more serious due to the compromised bone quality. Thus, this study is designed to determine if the novel TNZS implant with low elastic modulus and high strength can relieve the stress shielding phenomenon and improve the implant survival time.

In previous research, an implant with low elastic modulus enhanced new bone formation and suppressed bone resorption by the improvement of loading transmission. Cook et al. [20] concluded that the LTI carbon (14 GPa) implants with the direct implant-bone interface exhibited a greater potential for long-term successful performance compared to the aluminium oxide (350 GPa) implants. Lin et al. [21] found that Ti–7.5 Mo alloy with an elastic modulus of 65 GPa promoted new bone formation after being implanted in a rabbit femur. Sumitomo et al. [22] reported that Ti–29Nb–13Ta–4.6Zr alloy with an elastic modulus of 58 GPa minimised bone absorption and promoted bone reorganisation in the plate fixation of a tibia fracture in rabbit. Stoppie et al. [23] used animal and finite element studies to research the responses of peri-implant tissue to different elastic implants. The results showed that greater bone formation, thicker trabeculae and a higher rate of bone-to-implant contact were detected in the more elastic implants compared with the stiffer implants. However, all of these studies concentrated on normal bone tissue, with less of a focus on osteoporosis. In the present study, the osteoporotic rabbit model was induced by the combination of OVX and glucocorticoid treatment, which has previously been reported by several researchers [18],[24],[25]. This model has several advantages over other animal models: it is easy to obtain and care for, and the induction procedure is relatively short and uncomplicated with a high degree of reproducibility. The cylindrical implants (diameter of 2 mm and length of 6 mm) are suitable for implanting into the rabbit tibia. This model has been widely used to study bone ingrowth into implants and bone – implant interfaces [26]–[29], presenting a much faster bone turnover than other rodents. Moreover, in previous studies, our department has successful established the osteoporotic rabbit model [18]. All of these positive characteristics made the rabbit well-suited for this study.

After 4 weeks implantation, the bone formation around the implant was almost the same in both groups; however, higher BV and TMD were detected in the TNZS group compared with the TAV group at 12 weeks. Majority of researches [30]–[32] have suggested that bone formation is obviously affected by the surface morphology. To limit this effect, all of the implants were rubbed with 1,200-grit silicone paper to produce a uniform surface, and roughness measurements found no apparent differences in the surface of the implants in the two groups. Thus, the different results between the two groups were due to the different elastic modulus of the alloys and not the surface morphology of the alloys. With a high-elastic modulus, the TAV implant provided rigid fixation in the bone tissue, and little stress was transported to the peri-implant bone, which caused stress-shielding and bone absorption. For the TNZS implant with a low modulus, the stress was not only absorbed by the implant but also transported to the peri-implant bone, and this compatible stimulation of stress was beneficial for peri-bone formation and remodelling [33]. In finite element modelling studies, Hedia [34] indicated that a reduction of the elastic modulus of the implant could reduce the stress concentration in the cancellous and cortical bone by 15% and 16%, respectively; Simon et al. [35] suggested that the low-stiffness implants with a modulus closer to that of the surrounding trabecular bone would yield a more homogeneous stress distribution and less micro-motion at the interface with the bony bed. The reduced micro-motion and homogeneous stress distribution at the bone-implant interface was beneficial for bony ingrowth into the implant and for improving the stability of the implant. Hence, if the implants have enough strength to provide a stable fixation, we should use the implants with as low an elastic modulus as possible to minimise the mismatch between the bone and implant. Matching the modulus with the surrounding bone tissue is helpful in distributing stress uniformly, improving bone formation and lengthening the survival time of the implant.

It is believed that a strong bone-implant interface reduces harmful tissue strain and micro-motion between the bone and implant [36]–[39]. If the implant is not well integrated with the bone tissue, fibrous tissue forms between the bone and implant, which inhibits bone-implant osseointegration [40]. Hence, it is highly essential for the implant to integrate well with the adjacent bone tissue. In the present study, we evaluated the implant fixation strength with a pull-out test. Some researchers [41], feel that the pull-out test is more dependent on the properties of the peri-implant bone and less on the properties of the interface. In this study, we are focus on the different self-mechanical properties between the two implants, not the different surfaces of the implants. Meanwhile the pull-out test is generally used for cylindrical implants inserted in the proximal and distal portion of long bones [43]. The choice of the mechanical test depends on the clinically most significant failure mode of the tested device and its shape. However, which test best reflects the clinical failure of the implants remains a matter of debate [44]. The clinical environment is not being replicated in a pull-out test, and the test can only reflect a simplification of failure in the clinical situations. At 4 weeks, there was no difference in F_max_ between the TNZS and TAV groups; however, at 12 weeks after implantation, a significantly higher F_max_ was observed in the TNZS group compare with the TAV group. This result suggests that the TNZS implants have better stability and bone-implant interface than the TAV implants. Moreover, these biomechanical results were supported by histological observations, where a direct bone-to-implant contact with no fibrous tissue was detected in the TNZS group at 12 weeks, further proving that a strong bone-implant interface and osseointegration were achieved in the TNZS group.

It has been proposed that Al and V released by implants are potentially harmful and associated with the failure of clinical implantation [45]. By contrast, Nb and Zr have a smaller impact on cells and have never been shown or suggested as having short-term or long-term potentially adverse effects [46]–[48]. In our in vitro experiment, cell death was not found after seeding in both alloys, and stronger cell viability was shown in the TNZS group, which indicated that both TNZS and TAV have good biocompatibility, with TNZS having better one. Meanwhile, the higher ALP activity of the osteoblast were shown in the TNZS group at day 7, this was a hint that TNZS was more suitable for the osteogenic differentiation, bone formation and matrix mineralization, compare to TAV [49].

In the analyses, we did not use a histological index such as percentage of the bone-implant contact (PBC) or the percentage of new bone formation around the implants (PBF) to measure whether the bone had a good reaction to the implants as some differences could not be discovered in two-dimensional (2D) images; instead, we used radiological indexes like BV and TMD to show the quantity and the quality of the newly formed bone around the implants. Many studies have indicated that 3D results are more sensitive and accurate than 2D results and thus could give us an entire image of the situation around the implants; therefore, these 3D techniques were good ways to measure the bone formation [50].

In this study, rabbits were employed because of their size, temperament and the availability of genetically homogeneous strains [15],[51]. The drawbacks of this model for the assessment of implants are that only 6 implants can be evaluated in one rabbit and that the cylindrical implants are not recommended to be larger than 2 mm in diameter and 6 mm in length [15]. Despite this, the rabbit remains a very popular choice of species for studying bony ingrowth into implant and bone-implant interfaces. In this study, two types of implants were inserted simultaneously into the tibias of each animal. The interactive effects between the implants may slightly affect the results, and a control group may minimize this effect. Meanwhile, in the Micro-CT analysis, we use many methods to minimize the artefacts, such as making the implant at the centre of the scanning zone, using 360° strengthen scanning protocol and, enhancing the threshold of the bone. However, the artefacts can’t be fully avoided in Micro-CT scanning, and the results may be slightly affected by the artefacts. Nevertheless, the effect of the artifacts is almost equally to both groups, thus the conclusion will not be influenced.

## Conclusion

This study suggested that the novel Ti-24Nb-4Zr-7.9Sn alloy was particularly useful in biomedical applications. With good biocompatibility, ultra-low modulus and high strength, it is well-suited for orthopaedics, especially in osteoporosis.
